# Echocardiographic evaluation of right heart failure which might be associated with DNA damage response in SU5416-hypoxia induced pulmonary hypertension rat model

**DOI:** 10.1186/s12931-023-02501-7

**Published:** 2023-08-17

**Authors:** Meidan Kuang, Yilin Chen, Yue Xing, Min Du, Huazhuo Feng, Qifeng Yang, Dongmei Wen, Xuanyi Li, Kai Yang, Ziying Lin, Ning Lai, Qian Jiang, Shiyun Liu, Dansha Zhou, Wei Hong, Xin Fu, Wenju Lu, Tengteng Zhao, Jian Wang, Yuqin Chen

**Affiliations:** 1 State Key Laboratory of Respiratory Diseases, National Center for Respiratory Medicine, Guangdong Key Laboratory of Vascular Diseases, National Clinical Research Center for Respiratory Diseases, Guangzhou Institute of Respiratory Health, GMU-GIBH Joint School of Life Sciences, the First Affiliated Hospital of Guangzhou Medical University, Guangzhou Medical University, Guangzhou, 510120 Guangdong China; 2 Guangzhou Laboratory, Guangzhou International Bio Island, Guangzhou, 510320 Guangdong China; 3 The Jackson Laboratory, Bar Harbor, Maine, 04609 USA

**Keywords:** Right heart failure, Echocardiography, Pulmonary hypertension, Rats

## Abstract

Right heart failure is the leading cause of death in pulmonary hypertension (PH), and echocardiography is a commonly used tool for evaluating the risk hierarchy of PH. However, few studies have explored the dynamic changes in the structural and functional changes of the right heart during the process of PH. Previous studies have found that pulmonary circulation coupling right ventricular adaptation depends on the degree of pressure overload and other factors. In this study, we performed a time-dependent evaluation of right heart functional changes using transthoracic echocardiography in a SU5416 plus hypoxia (SuHx)-induced PH rat model. Rats were examined in 1-, 2-, 4-, and 6-week using right-heart catheterization, cardiac echocardiography, and harvested heart tissue. Our study found that echocardiographic measures of the right ventricle (RV) gradually worsened with the increase of right ventricular systolic pressure, and right heart hypofunction occurred at an earlier stage than pulmonary artery thickening during the development of PH. Furthermore, sarco-endoplasmic reticulum calcium ATPase 2 (SERCA2), a marker of myocardial damage, was highly expressed in week 2 of SuHx-induced PH and had higher levels of expression of γ-H2AX at all timepoints, as well as higher levels of DDR-related proteins p-ATM and p53/p-p53 and p21 in week 4 and week 6. Our study demonstrates that the structure and function of the RV begin to deteriorate with DNA damage and cellular senescence during the early stages of PH development.

**Supplementary Information:**

The online version contains supplementary material available at 10.1186/s12931-023-02501-7.

## Introduction

Pulmonary hypertension (PH) is a syndrome characterized by a gradual increase in pulmonary vascular pressure and resistance, ultimately leading to right heart failure, which is the primary cause of death [1, 2]. Studies have shown that decreased right ventricular ejection fraction, rather than increased pulmonary vascular resistance, is more closely related to mortality in PH patients [3]. Despite the recent focus on right heart function in PH development, treatments still mainly target the vasodilation of pulmonary arteries.

Assessing the right ventricular ejection fraction is more meaningful in evaluating the prognosis of PH [4]. Additionally, right heart fibrosis and myocardial hypertrophy have been observed in both human and rat PH models, indicating that changes in right heart function play a crucial role in determining the prognosis of PH patients [5]. As the disease progresses, compensatory alterations in the right ventricle (RV) linked to increased pulmonary pressure may lead to right heart failure [6]. Therefore, developing a model of right heart failure is necessary. The disease progression and pathological alterations of the SU5416 plus hypoxia (SuHx)-induced PH animal model resemble those in patients with severe PH [7]. It remains unclear how right ventricular function and shape change over time as PH develops and whether these changes impact the disease’s progression and underlying causes.

DNA damage response (DDR) occurs physiologically in the part of cell metabolism to maintaining genomic stability or could be induced by genotoxic agents [8, 9]. Single strand breaks (SSBs) are the most common type of DNA damage, and if not repaired properly, can lead to double-strand breaks (DSBs). Besides, both endogenous and external factors can cause DSBs including accumulated genetic damage, hypoxia, and inflammation. Studies have shown that DNA damage, particularly DSBs, can cause the phosphorylation of ataxia telangiectasia mutated protein (ATM), which activates downstream signaling pathways and phosphorylation of p53, leading to cell cycle arrest, cell senescence, cell hyperplasia, flattening, and functional decline [10]. Myocardial cell senescence has not been extensively studied, but research on aged hearts has shown that hypertrophic aging hearts have increased myocardial nuclei and connective tissue deposition [11]. In this study, we investigated the structural and functional changes of the right heart associated with the SuHx-induced PH rat model during disease progression and tested some proteins associated with DDRs.

## Materials and methods

### Establishment of SuHx-induced PH rat model

Adult male Sprague Dawley rats (6–8 weeks old, 190–220 g) were used for the experiments. To induce PH, the rats were given a single subcutaneous injection of SU5416 (20 mg/kg) purchased from MedChemExpress (Cat# HY-10,374/CS-1225, Long Branch, NJ, USA). Subsequently, the rats were exposed to hypoxia condition (10% O_2_) in a chamber for 3 weeks followed by a return to normoxia for 3 weeks. The rats in the control groups were injected with equal volumes of vehicle (DMSO) and kept under normoxic conditions. The rats were randomly divided into SuHx groups and control groups (CTL), including the 1-week SuHx group (n = 6), 1-week control group (n = 5), 2-week SuHx group (n = 6), 2-week control group (n = 7), 4-week SuHx group (n = 6), 4-week control group (n = 5), 6-week SuHx group (n = 7), and 6-week control group (n = 7). All procedures were approved by the Animal Care and Use Committee of Guangzhou Medical University. Animals were housed at 24 °C in a 12-h light-dark cycle. Food and water were accessible freely.

### Echocardiographic measurements

The rats were anesthetized with isoflurane, with a 5% concentration for anesthesia induction lasting 1–2 min and followed by a maintenance concentration of 2%. Then the rats were transferred onto a heating plate to maintain their body temperature at 37 °C. Their heart rates were continuously monitored above 300 bpm after inhalation induction. After shaving the precordium area, echocardiography of the heart was performed using a Vevo 2100 Imaging System equipped with an MS250 (13–24 MHz) linear array transducer (FUJIFILM Visual Sonics, Toronto, Canada). The parameters for right heart function, including tricuspid annular plane systolic excursion (TAPSE), RV end-diastolic free-wall thickness (RVEDWT), RV end-systolic free-wall thickness (RVESWT), RV end-diastolic diameter (RVEDD), RV end-systolic diameter (RVESD), pulmonary acceleration time (PAT), and the ratio of PAT/pulmonary ejection time (PAT/PET), were measured. The parameters for left heart function included left ventricular fractional shortening (LVFS) and left ventricular ejection fraction (LVEF). RV free-wall thickness fractional thickening (RVWTFT) and RV diameter fractional shortening (RVDFS) were calculated using the following formulas.

$$\text{R}\text{V}\text{W}\text{T}\text{F}\text{T}=(\text{R}\text{V}\text{E}\text{S}\text{W}\text{T} - \text{R}\text{V}\text{E}\text{D}\text{W}\text{T})/\text{R}\text{V}\text{E}\text{S}\text{W}\text{T}$$

$$\text{R}\text{V}\text{D}\text{F}\text{S}=(\text{R}\text{V}\text{E}\text{D}\text{D} - \text{R}\text{V}\text{E}\text{S}\text{D})/\text{R}\text{V}\text{E}\text{D}\text{D}$$

### In vivo hemodynamic measurements

All rats were anesthetized with intraperitoneal injection of pentobarbital sodium (30 mg/kg) and placed on a heating pad. Hemodynamic measurements were performed under normoxic conditions at room temperature. After locating the right jugular vein, a polyvinyl catheter was inserted into the RV to measure right ventricular systolic pressure (RVSP), as the pulmonary artery systolic pressure (PASP) was undetectable. The skin along the right paraxial line was cut to expose the third and fourth ribs. Subsequently, the third and fourth ribs were removed to expose the heart, and the aortic root was dissociated to approximately 0.2 cm. A flowmeter probe was then set in the dissected aortic segment to record the real-time blood flow waveforms using an MP150 physiological recorder Acknowledge Software system. Finally, the needle attached to the baroreceptor was inserted into the RV, and the RV time-pressure waveform was acquired by the software. The system required thirty seconds to reach equilibrium.

### RV hypertrophy and tissue harvest

After perfusing the animals with phosphate-buffered saline to remove peripheral blood cells, the lungs and hearts were harvested. Using microscissors under an integrated microscope, the RV was carefully separated from the outlet of the pulmonary artery along the edge of the free wall of the RV. The remaining part was considered as the left ventricle plus septum (LV + S). RV hypertrophy was assessed by calculating the ratio of RV wet weight to LV + S wet weight (RV/LV + S) and the ratio of RV wet weight to body weight (RV/BW).

### Morphological and histological analyses

After the heart and lung were isolated, the tissue was fixed with 10% neutral buffered formalin for 48 h before paraffin embedded, and were followed by sectioned into slices with a thickness of 5 μm. Subsequently, the morphological and histological analyses were performed by hematoxylin and eosin (H&E) staining and masson’s trichrome staining.

#### Pulmonary vessel medial wall thickness and medial wall area

The evaluation of pulmonary vessel wall thickness primarily relies on two indices: (1) ratio of medial wall thickness, which is calculated by subtracting the inner circumference from the outer circumference and then dividing it by the outer circumference; (2) ratio of medial wall area, which is determined by subtracting the vessel lumen area from the total vessel area and then dividing it by the total vessel area. The circumference and area were measured by CaseViewer (3DHISTECH, Budapest, Hungary).

#### Right ventricle hypertrophy

The cross-sectional area was used to assess the hypertrophy of the RV. The cross-sectional area of RV myocardial cells was randomly measured using the iViewer 6.0 slicing analysis software (Unic Technologies Inc., Beijing, China) on H&E-stained tissue sections. For each rat, a total of 50 circular-shaped myocardial cells were selected for area measurement, and the average value was calculated.

#### Right ventricle fibrosis

Quantitative right ventricle fibrosis analysis was carried out using ImagePro Plus 6 software to perform optical density statistics on Masson-stained sections of right ventricle tissue. The measured value is expressed as optical density/measured area.

### Immunofluorescence staining

The heart tissues slices were also used for immunofluorescence staining. To prepare the slices, the heart sections were dewaxed and dehydrated, followed by antigen retrieval in boiling EDTA antigen retrieval buffer. Subsequently, the slides were incubated overnight at 4 °C with primary antibodies (α-SMA, Servicebio, Cat. #GB111364; p53, Santa Cruz, c-126; γ-H2AX, Abcam, Cat# ab81299). Afterward, the slides were washed three times with PBS and incubated with secondary antibodies (FITC, EarthOx, Cat. #E031210; Cy3, EarthOx, Cat. #E031610) for 2 h at room temperature. Negative controls were included without the use of primary antibodies. The nuclei were counterstained with DAPI. Imaging was performed using a laser-scanning confocal fluorescence microscope (Nikon, Tokyo, Japan). Four random microscopic fields were selected from each slide to calculate the relative fluorescence intensity, and an average value was determined for each rat.

### Western blot and analysis

To extract proteins, the right ventricular tissue of rats was sonicated in RIPA lysis buffer (Beyotime, Shanghai, China) supplemented with a protease inhibitor cocktail and PMSF fluoride. Protein expression was measured by immunoblotting, as previously described. Protein lysates were quantified using the bicinchoninic acid protein assay (Pierce, Rockford, IL, USA). Protein aliquots were denatured by adding 5×SDS-PAGE loading buffer and heating at 100 °C for 5 min, and then resolved by 10% SDS‒PAGE. Separated proteins were transferred onto polyvinylidene difluoride membranes (pore size 0.45 µM; Bio-Rad, Hercules, CA, USA). The membranes were blocked with 5% nonfat dry milk in Tris-buffered saline containing 0.2% Tween 20 and then probed with affinity-purified rabbit polyclonal antibodies specific for SERCA2, γ-H2AX, p-ATM, p53, p-p53, and p21, or mouse monoclonal antibodies for GAPDH. The antibodies against SERCA2, γ-H2AX, and p-ATM were purchased from Abcam (Cat# ab150435; Cat# ab81299; Cat# ab81292). The antibodies against GAPDH, p-p53, p53, and p21 were purchased from Santa Cruz (Cat# sc-365,062; Cat# sc-51,690; Cat# sc-126; Cat# sc-6246).

### Statistical analysis

All data were analyzed by SPSS 16.0 statistical software, and the experimental results are expressed as the mean ± standard error (mean ± S.E.M.). The differences between the model group and the control group were analyzed and counted by student’s t tests. Nonparametric Mann-Whitney U test was used for the data which was not uniform or a normal distribution. Spearman’s correlation and linear regression were utilized to analyze correlation between RVSP and echocardiographic measurements.

## Results

### Hemodynamic evaluation of the SuHx-induced PH at weekly time point

**Fig. 1:**
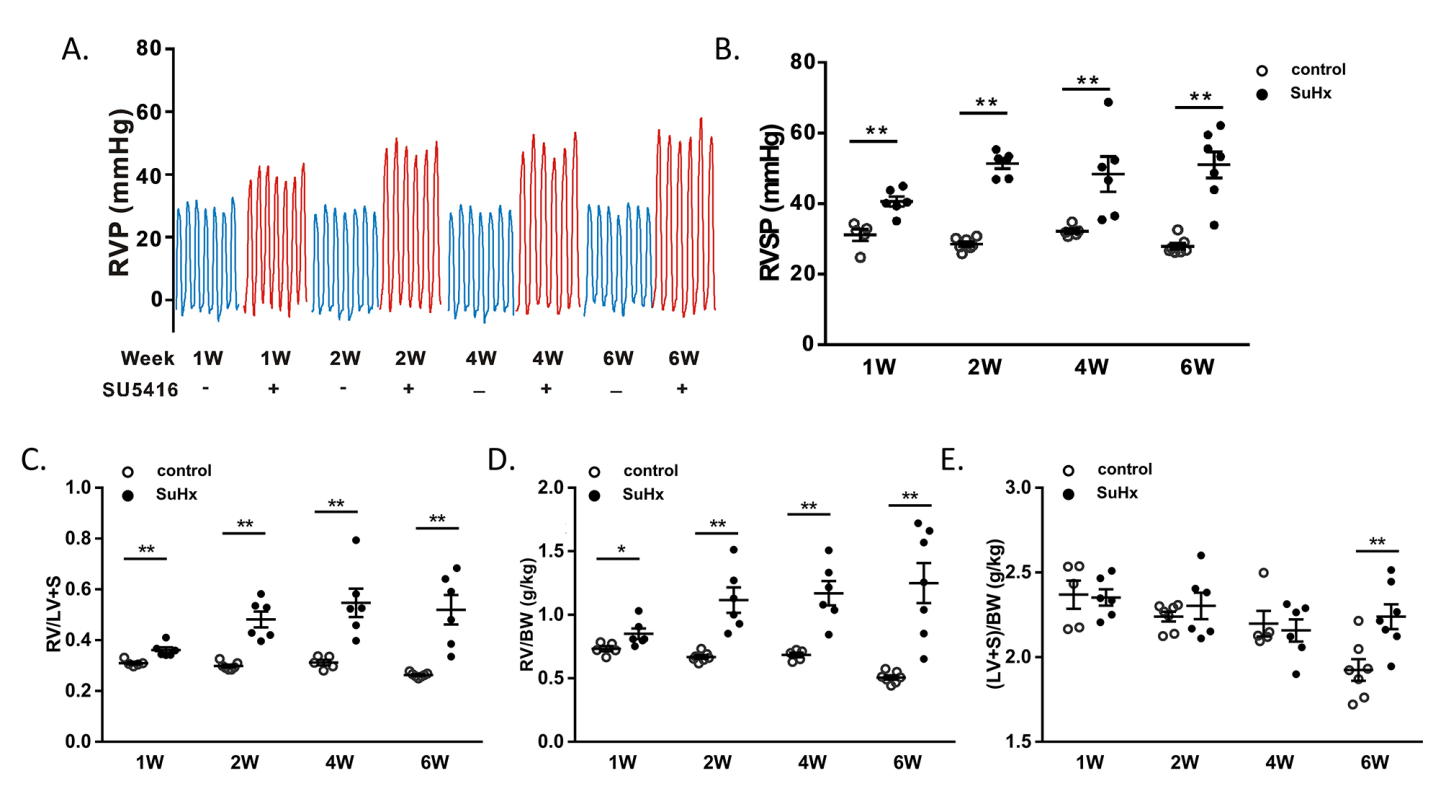
Effects of SU5416 and hypoxia on the rats’ right ventricular (RV) pressure and hypertrophy. (**A**) Line chart showing the changes in RVP of SuHx rats and control rats in 1- to 6-week. (**B**) Scatter dot plot showing the RVSP of two groups in 1-, 2-, 4- and 6-week. (**C**) Comparisons of the ratio of RV weight to left ventricular (LV) and interventricular septum [RV/(LV + S)]. (**D**) RV to BW ratio (RV/BW). (**E**) LV and S to BW ration [(LV + S)/BW]. Data were presented as mean ± S.E.M., control group n = 5–7, SuHx group n = 6–7, *P < 0.05, **P < 0.01, compared with control group. W: week

**Fig. 2:**
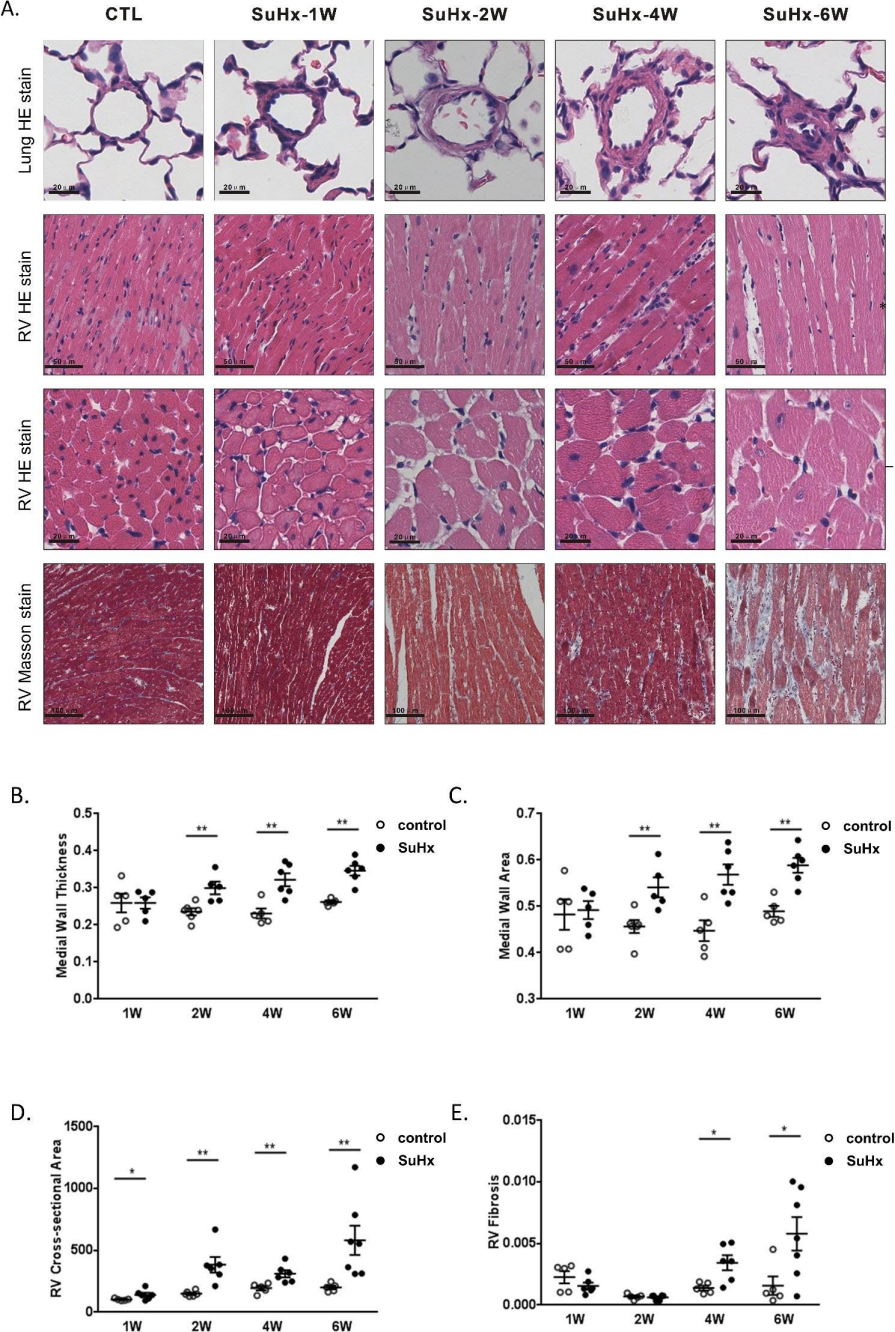
Changes of pulmonary vascular medial wall thickness and RV lesions in SuHx rats. (**A**) Representative photomicrographs of hematoxylin and eosin (H&E) stain of pulmonary vessel cross sections, H&E stained and masson’s trichrome stain of RV sections. (**B, C**) Scatter dot plot showing the comparison of vessel wall thickness and vessel wall area of vessels (outside diameter > 50 μm) between the CTL and SuHx groups. (**D, E**) Scatter dot plot showing the comparison of cross-sectional area of right ventricle cardiomyocyte and fibrosis between two groups. Data were presented as mean ± S.E.M., control group n = 5–7, SuHx group n = 6–7, *P < 0.05, **P < 0.01, compared with the control group. W: week

To confirm the development of PH, we measured the right ventricular pressure (RVP) waveform in rats using a Millar catheter inserted in the jugular vein (Fig. 1A). We found no significant differences between the control groups. Compared to the control group of rats, the RV systolic pressure (RVSP) was significantly increased in SuHx-induced PH rats in the first, second, fourth, and sixth weeks, peaking in the second week (RVSP = 51.3 ± 1.4 mmHg) and then stabilizing. There were no significant differences in RVSP between the SuHx groups in week 1 to week 6 (Fig. 1B). Similarly, the relative RV mass measured at autopsy by RV/(LV + S) was significantly elevated at first, second, fourth, and sixth weeks in the SuHx groups compared to controls (Fig. 1C, D and E). However, unlike RVSP, RV/(L + S) in the SuHx groups showed an upward trend in week 4 to 6. In addition, to observe whether SU5416 affected LV + S, we normalized the mass of RV and LV + S by body weight (BW). RV/BW was significantly elevated compared with the control group, as was RV/(L + S). (LV + S)/BW in the SuHx group showed no significant change in week 1, week 2 and week 4, but significantly increased in 6-week (*P* < 0.01). (Fig. 2D and E)

### SuHx-induced PH exhibited increased pulmonary arterial remodeling, RV hypertrophy and heart fibrosis

The degree of pulmonary vascular thickening and right ventricular remodeling were assessed by H&E staining, and right ventricular fibrosis was evaluated using masson’s staining (Fig. 2A). In the SuHx groups, there was a gradual thickening of the pulmonary vascular wall over time, and occlusion was observed only in week 6. Compared to the control groups, the SuHx-induced PH rats showed no significant difference in medial wall thickness and medial wall area in 1-week, but they significantly increased in 2-week and peaked in 6-week (Fig. 2B and C). Similarly, compared to the control groups, the SuHx groups exhibited progressive myocardial hypertrophy, with an increase in the cross-sectional area of right ventricular cardiomyocytes observed in 1-week and reaching its maximum in 6-week (Fig. 2B, D). Masson’s staining revealed that in the SuHx groups, the degree of fibrosis in the right heart remained unchanged in week 1 and week 2, but it began to increase in week 4 and reached its highest level in week 6 (Fig. 2B, E).

### Structural changes in the right heart of SuHx-induced PH rats

**Fig. 3:**
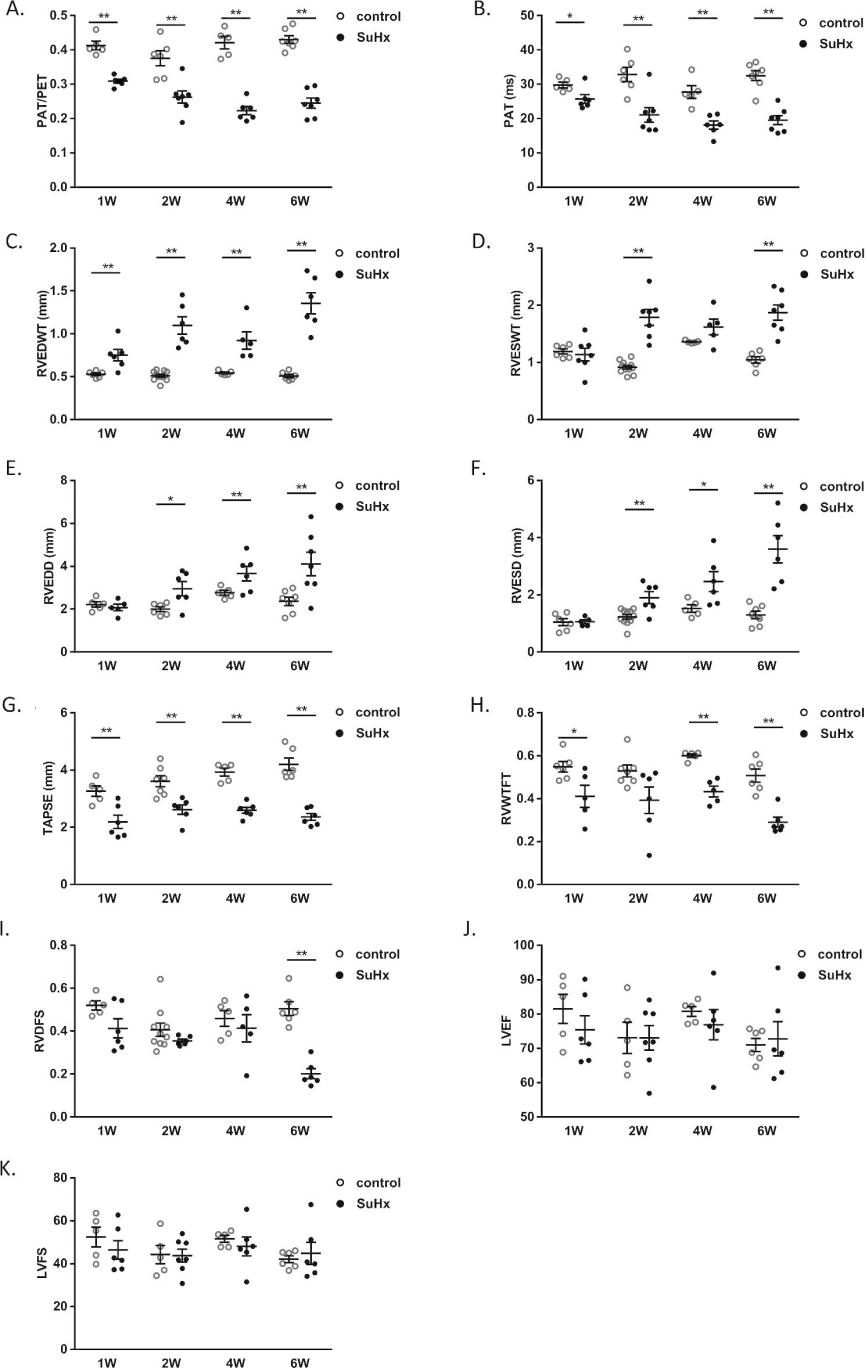
Dynamic changes in echocardiographic measurements of SuHx rats. Scatter dot plots showing the echocardiographic indexes including PAT/PET ratios (**A**), PAT (**B**), RVEDWT (**C**), RVESWT (**D**), RVEDD (**E**), RVESD (**F**), TAPSE (**G**), RVWTFT (**H**), RVDFS (**I**), LVEF (**J**), and LVFS (**K**) the two groups. Data were presented as mean ± S.E.M., control group n = 5–7, SuHx group n = 6–7, *P < 0.05, **P < 0.01, compared with the control group. PAT: Pulmonary acceleration time; PAT/PET: the ratio of PAT/pulmonary ejection time; RVEDWT: RV end-diastolic free-wall thickness; RVESWT: RV end-systolic free-wall thickness; RVEDD: RV end-diastolic diameter; RVESD: RV end-systolic diameter; TAPSE: Tricuspid annular plane systolic excursion; RVWTFT: RV free-wall thickness fractional thickening; RVDFS: RV diameter fractional shortening; LVEF: left ventricular ejection fraction; LVFS: left ventricular ejection fraction; W: week

As shown in Fig. 3A and B, PAT/PET and PAT began to decrease in the first week, and reach their lowest level in week 4. RVEDWT increased significantly in week 1 and peaked in week 6 (Fig. 3B, C). RVSDWT showed no significant change in week 1, while it increased significantly in week 2, and reached its highest value in week 6 (Fig. 3B, D). RVEDD and RVESD did not change in week 1, but began to increase in week 2 and peaked in week 6 (Fig. 3B, E, F).

### SuHx-induced PH exhibited decreased right heart function but normal left heart function

**Fig. 4:**
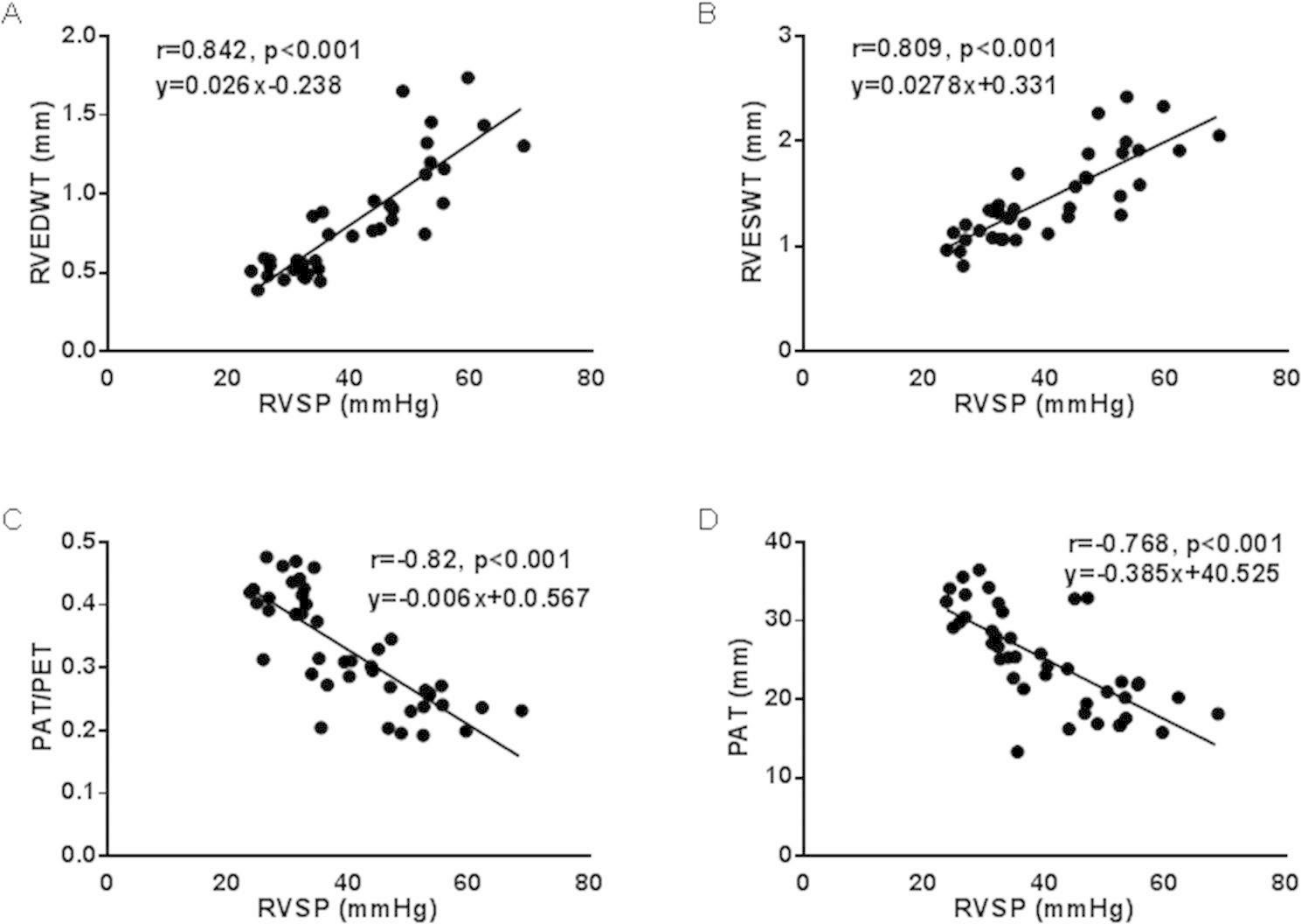
Correlation analysis of RVSP and echocardiographic measurements. RVEDWT (**A**), RVESWT (**B**), PAT/PET ratios (**C**) or PAT (**D**) measured by echocardiography in rat models of PH. Linear correlation between RVSP and RVEDWT, RVESWT, PAT/PET ratios or PAT were demonstrated

Compared to the control groups, TAPSE in the SuHx rats showed a significant decrease in week 1, but there was no clear trend of worsening or improvement (Fig. 3B, G). RVWTFT started to decrease in week 1 and reached its lowest level in week 6 (Fig. 3B, H). RVDFS did not show significant changes in week 1, week 2 and week 4, but significantly decreased in week 6 (Fig. 3B, I). However, in SuHx rats, LVEF and LVFS, which are indicators related to left heart systolic function, did not show significant changes in each stage compared to the normal group (Fig. 3B, J and K). The correlation between RVSP and echocardiographic parameters was then analyzed. There was a strongly positive correlation between RVEDWT and RVSP (r = 0.842, Fig. 4A), as well as between RVESWT and RVSP (r = 0.809, Fig. 4B). Meanwhile, there was a clear negative correlation of PAT (r = -0.82, Fig. 4C) and PAT/PET (r = -0.768, Fig. 4D) with RVSP.

### The expression of SERCA2 in the right ventricles of SuHx-induced PH rats

**Fig. 5:**
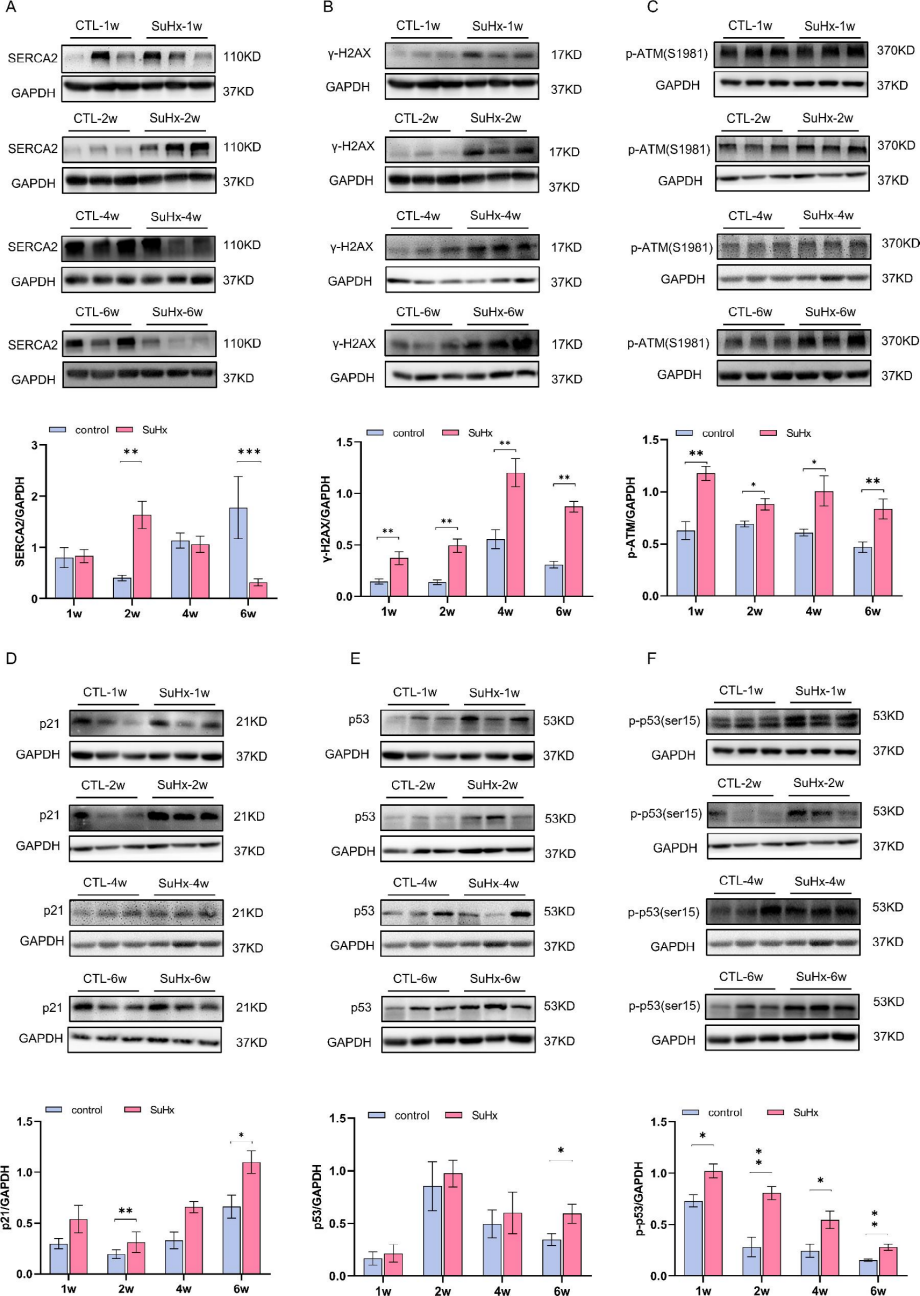
Changes in expression level of myocardial damage and DNA damage related proteins in RV tissue of SuHx rats. (**A**) Representative blots for (A) SERCA2, (**B**) γ-H2AX, (**C**) p-ATM, (**D**) p21, (**E**) p-p53, p53 and GAPDH. Mean intensity for SERCA2, γ-H2AX, p-ATM, p21, (**F**) p-p53 and p53 blots relative to GAPDH. Data were presented as mean ± S.E.M., control group n = 5–7, SuHx group n = 6–7, *P < 0.05, **P < 0.01, compared with the control group. The gel images shown are cropped from the full-length blot images which were shown in additional file 1. W: week

The expression of sarco-endoplasmic reticulum calcium ATPase 2 (SERCA2), a functional protein in myocardial cell, showed a trend of initial increase and followed by subsequent decrease in SuHx-induced PH rats. Compared with the control groups, SERCA2 protein expression did not change significantlyin week 1 but increased significantly in week 2, showed no significant difference in week 4, and decreased significantly in week 6 in the SuHx groups. (Fig. 5A and B)

### The increased injury repair response in the right ventricles of SuHx-induced PH rats

**Fig. 6:**
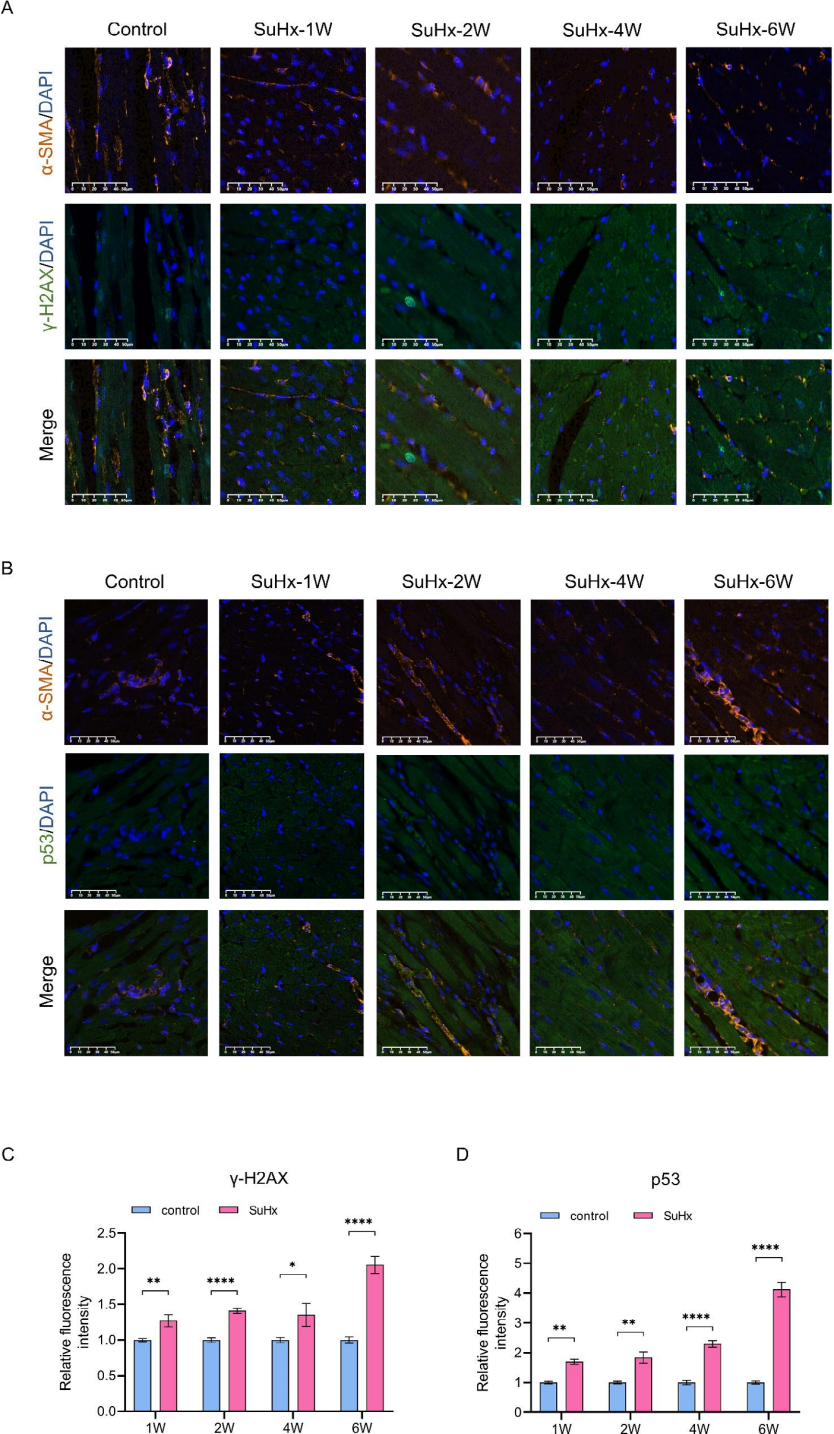
Evaluation of DNA damage in RV tissue during the process of PH in SuHx-induced rat model. (**A** and **B**) Representative images of the immunofluorescent double staining of specific smooth muscle marker α-smooth muscle actin (α-SMA, red), γ-H2AX (or p53, green) and DAPI (blue). The summary data presented the relative fluorescent intensity of γ-H2AX-positive (**C**) or p53-positive (**D**) RV cardiomyocytes, which were normalized to the control group. Data were presented as mean ± S.E.M., W: week

To assess the development of DNA damage in the right ventricular tissue, we detected the expression level of DDRs related proteins. The expression of γ-H2AX was significantly increased in SuHx rats compared to the control group at all time points (Fig. 5A and B, and Fig. 6A C). In addition, the expression levels of p-p53, p-ATM, and p21 were increased in the right ventricular tissue of SuHx rats. The expression of p-ATM was also significantly increased in the right ventricular tissue of SuHx rats at all developing phases. (Fig. 5B, E, and F). The expression of total p21 protein in the right ventricular tissue was not significantly different in week 1 and week 2, but increased significantly compared to the control group in week 4 and week 6 (Fig. 5G). Similarly, the expression of total p53 protein showed no significant difference between the SuHx group and the control group in week 1, week 2 and week 4, but increased significantly in week 6 (Fig. 5I and J). Whereas immunofluorescent staining showed that p53 was up-regulated since week 1 and persisting throughout the disease (Fig. 6B and D). The expression of p-p53 was also significantly increased in RV tissue of SuHx rats at all evaluated timepoints (Fig. 5B, I, and J).

## Discussion

This study presents a comprehensive evaluation of the dynamic changes in the structure and function of the right heart in SuHx-induced PH rats using pathology and echocardiography. Additionally, it primarily assesses the level of DNA damage in RV along with increased pulmonary vascular resistance. We observed that the right ventricular hypertrophy and dysfunction (as indicated by increased RV cross-sectional area and decreased PAT/PET and TAPSE on echocardiography) in SuHx rats were evident from the first week of disease progression, accompanied by an increase in RVSP. However, there was no discernible alteration in pulmonary vascular remodeling or left heart function at this stage. Interestingly, the right ventricular diameter and cardiac fibrosis were not significantly altered, suggesting that the right heart is particularly susceptible to increasing afterload. It is known that the expansion of the RV is a compensatory or decompensated lesion caused by sustained afterload rise or moderate to severe increase in pulmonary arterial pressure [12]. Consistent with Spyropoulos and colleague’s measurements[13], our results showed that the correlation was highly significant between RVSP and echocardiographic parameters evaluating right heart function (reflected by RV free-wall thickness and PAT/PET). Moreover, the increase of myocardial cell cross-sectional area of RV was essentially consistent with the RVSP trend at each stage of PH progression.

In addition to compensatory changes such as an increased right ventricular diameter and right heart hypertrophy, the SuHx group also exhibited decompensated alterations in week 4, including increased right ventricular fibrosis and a significant decline in RVWTFT. Although the RVSP in SuHx rats stopped rising in week 6, vascular obstruction and increased artery wall thickness still persisted. During this period, pulmonary vascular remodeling was the primary factor contributing to the increase in RVSP. Right ventricular fibrosis and most of echocardiographic markers in PH rats continued to increase in week 6, while RVDFS began to decline. Interestingly, the morphological and functional changes in the right heart in SuHx groups occurred earlier than the morphological changes in pulmonary vessels, indicating that factors beyond right cardiac afterload could impact the right heart function in the early stages of PH.

To assess changes in myocardial function in the RV, we analyzed the expression of SERCA2, which is a critical component of cardiomyocyte excitation-contraction coupling. During the action potential of mammalian cardiomyocytes, calcium ions enter the cells via L-type calcium channels, and calcium ions from the sarcoplasmic reticulum subsequently flow into the cells in the form of calcium released from calcium, leading to an increase in intracellular calcium ions and cell contraction. During diastole, SERCA pumps 70% of intracellular calcium ions back to the sarcoplasmic reticulum [14]. Therefore, SERCA activity is crucial for maintaining heart systolic function and is one of the key regulators of cardiac systole and diastole [15]. Studies using lentivirus and adenovirus to overexpress SERCA2 have demonstrated a causal relationship between heart failure and reduced function resulting from decreased SERCA activity [16]. In the SuHx groups, SERCA2 levels displayed an increasing trend in week 1 that was not statistically significant, a significant increase in expression in week 2, a non-statistically significant decline in week 4, and a significant decrease in week 6. This suggests that SERCA2 was initially elevated in a compensatory manner during the early stages of PH but decreased as the disease progressed.

The potential underlying mechanisms of the morphological and functional changes observed in the RV in SuHx-induced PH were further investigated in this study. DNA damage to cardiomyocytes was examined, as it has been recently implicated in PH pathogenesis and heart failure [17–19]. Various factors, including endogenous and exogenous genotoxic substances can induce DNA damage and repair response [20]. Unhealed single-strand breaks can lead to double-strand breaks and increased γ-H2AX biomarker expression [19, 21]. DNA damage triggers DNA damage responses that determine cell fate, with DSBs causing ATM phosphorylation [22, 23] and downstream signaling that promotes cell cycle arrest, hypertrophy, and functional decline [24, 25]. Cellular senescence, which can be caused by DNA damage, is a permanent form of cell cycle arrest and is related to pulmonary function and disease prognosis [26]. Recently some protein biomarkers of cellular senescence has been found to be clinically related to pulmonary function and disease prognosis and pathological changes on pulmonary fibrosis[27, 28], and basically regulated the p38/p53/p21 signaling pathway[29]. Meanwhile, DNA damage eventually leads to either apoptotic cell death of endothelium in PH[30] or survival of cells with genomic abnormalities, causing dysfunction, hypertrophy, and senescence[31], which are consistent with the previously observed pathological and functional changes in the RV. Accordingly, we observed an elevated level of DNA damage with increased expression of γ-H2AX throughout all stages of SuHx-induced PH. At the 4- and 6-week time points, we observed elevated levels of DDR-related proteins, including p-ATM, p53/p-p53, and p21. DNA damage was present in the early stages of SuHx rats and persisted over time, with expression of the cell cycle tissue arrest-associated protein p21 increasing as the disease progressed. The changes in right heart function and DNA damage coincided with an increase in RVSP, indicating that the alterations in right heart function may not be solely due to increased afterload, but also to the early onset of myocardial DNA damage. These factors may cumulatively impair right heart function and accelerate the formation of myocardial fibrosis. Right heart failure in the progression of PH may result from multiple factors, and the specific mechanisms need to be further explored in future studies.

In summary, we systematically evaluated the dynamic changes in the structure and function of the RV using the SuHx-induced PH rat model, which is characterized by gradually increased RVSP and pulmonary vascular remodeling. We observed that structural changes and dysfunction of RV could occur earlier than pulmonary vascular remodeling in the progression of PH, which may be associated with DNA damage in the early cardiac phase, and placed an emphasis on early protection for the right heart to delay the right heart failure.

## Conclusions

We observed dynamic changes in the structure and function of the RV during PH development using the SuHx-induced PH rat model. Notably, echocardiographic measures of RV function and pulmonary hemodynamics were strongly correlated. As PH progressed, we noted a deterioration in myocardial hypertrophy and systolic function of the right heart even in the earlier stages, possibly due to DNA damage response and cellular senescence. These findings suggest that DNA damage may play a crucial role in the pathogenesis of PH and right heart failure. Further studies are needed to elucidate the specific mechanisms involved.

### Electronic supplementary material

Below is the link to the electronic supplementary material.

Supplementary Material 1

## Data Availability

The data that support the findings of this study are available from the corresponding author upon reasonable request.
