# Supplementary material for: Echocardiographic evaluation of right heart failure which might be associated with DNA damage response in SU5416-hypoxia induced pulmonary hypertension rat model
Source: Respir Res. 2023 Aug 17;24:202. doi: 10.1186/s12931-023-02501-7 (PMC10433698; doi:10.1186/s12931-023-02501-7)
Supplement: Supplementary file 1 — Supplementary Material 1 [file 12931_2023_2501_MOESM1_ESM.pdf]

Full Western blot images of Figure 5A

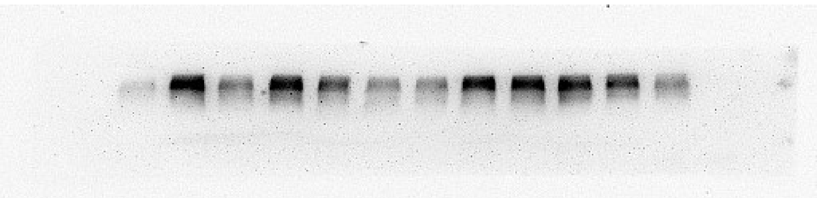

SERCA 2 (week 1)

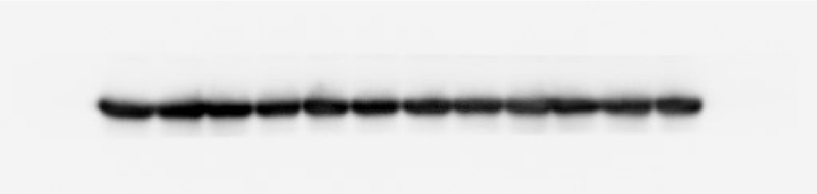

GAPDH (week 1)

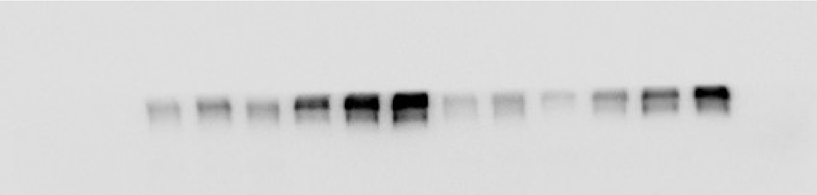

SERCA 2 (week 2)

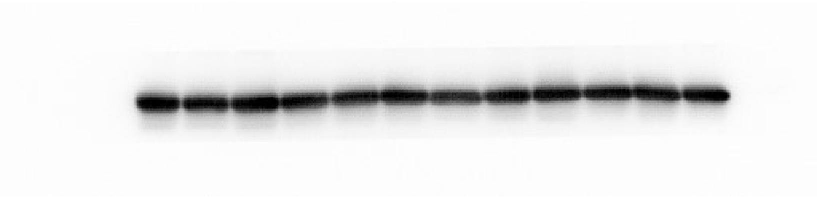

GAPDH (week 2)

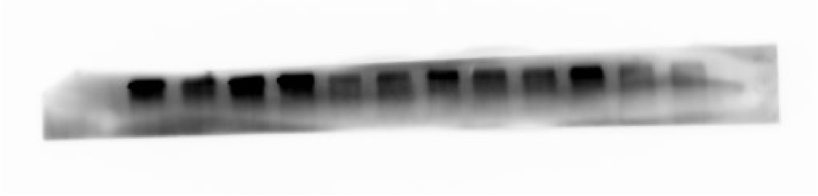

SERCA 2 (week 4)

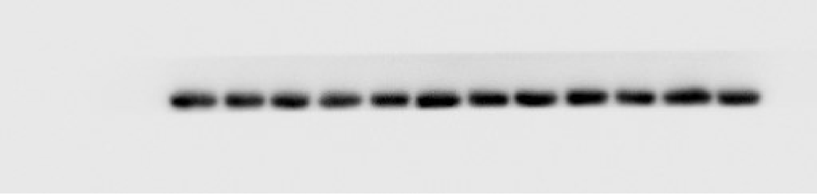

GAPDH (week 4)

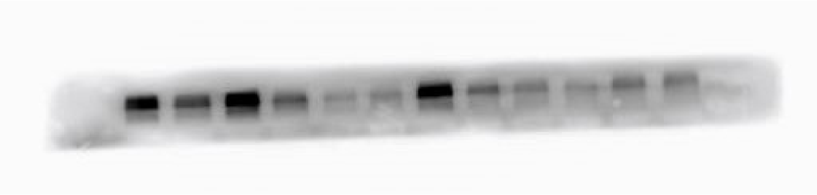

SERCA 2 (week 6)

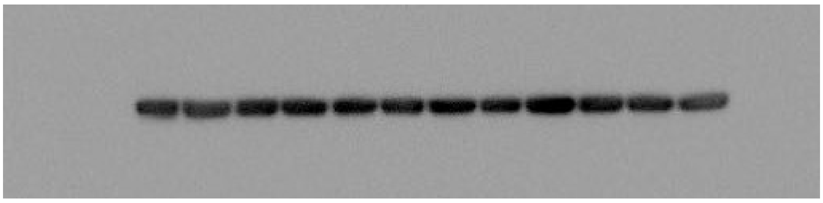

GAPDH (week 6)

Full Western blot images of Figure 5B

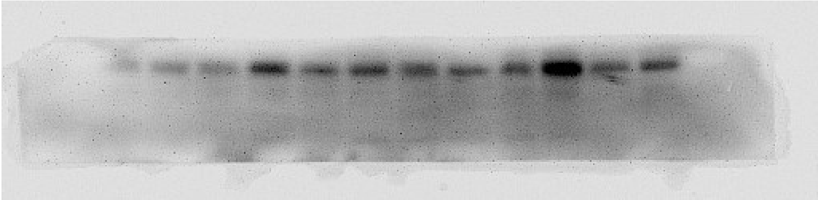

γ-H2AX (week 1)

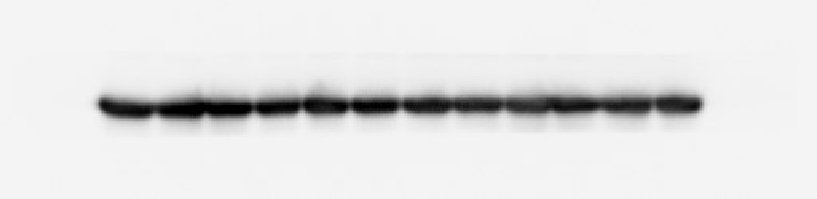

GAPDH (week 1)

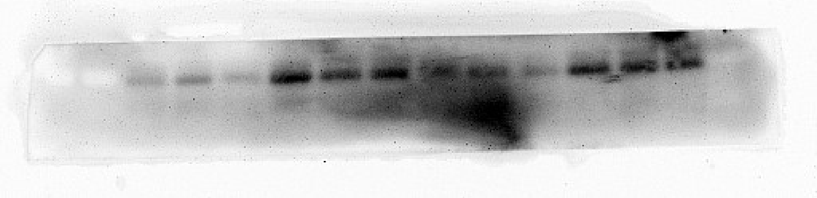

γ-H2AX (week 2)

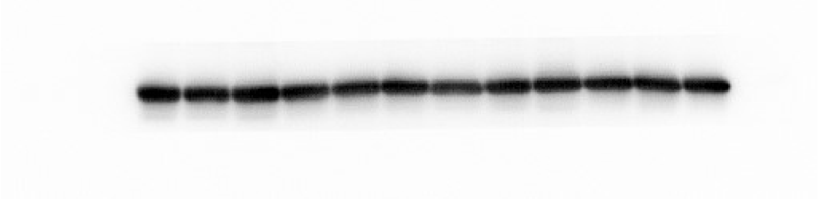

GAPDH (week 2)

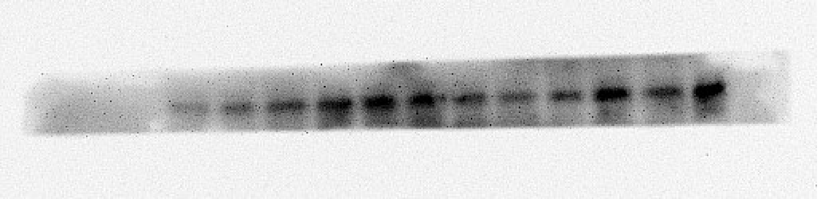

γ-H2AX (week 4)

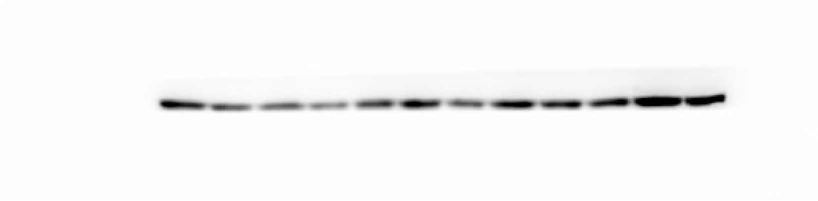

GAPDH (week 4)

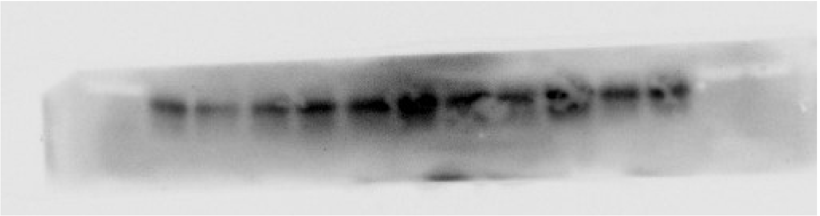

γ-H2AX (week 6)

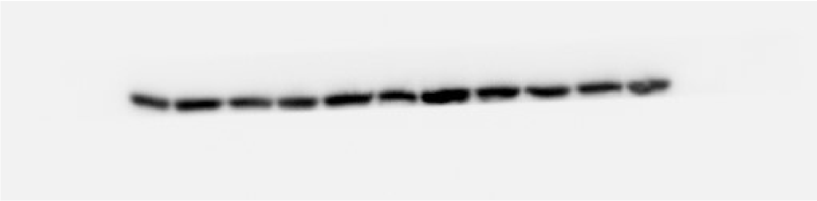

GAPDH (week 6)

Full Western blot images of Figure 5C

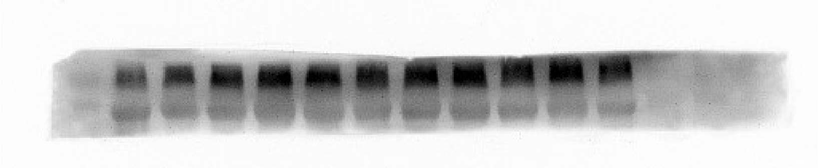

p-ATM (week 1)

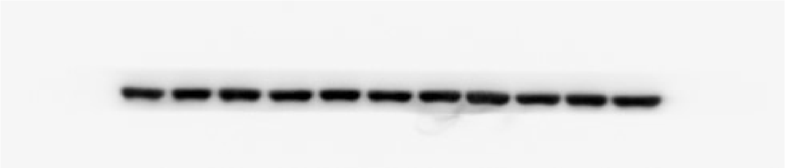

GAPDH (week 1)

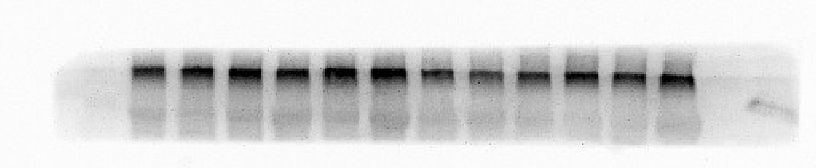

p-ATM (week 2)

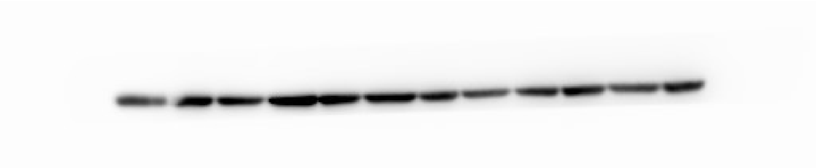

GAPDH (week 2)

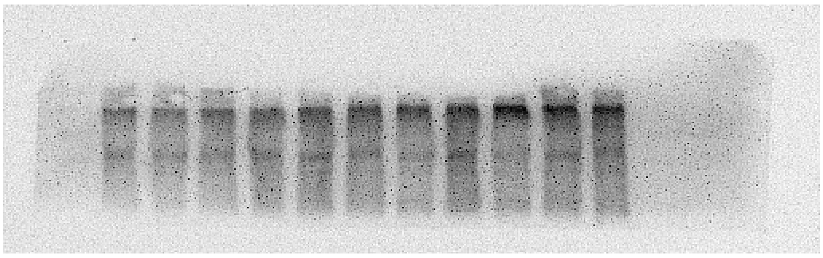

p-ATM (week 4)

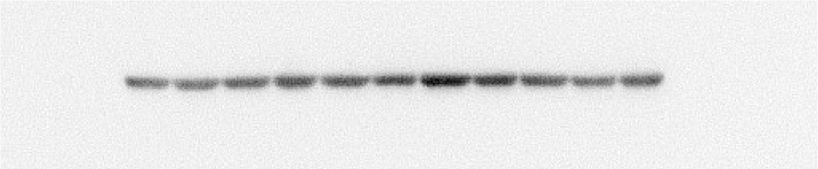

GAPDH (week 4)

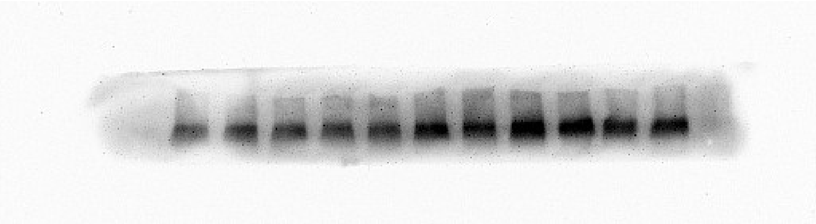

p-ATM (week 6)

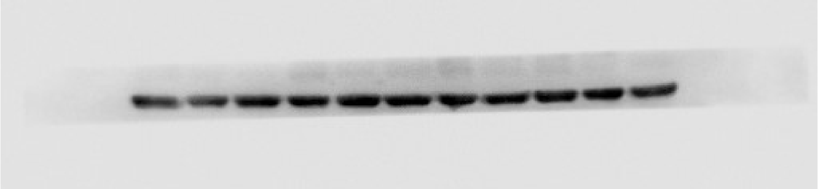

GAPDH (week 6)

Full Western blot images of Figure 5D

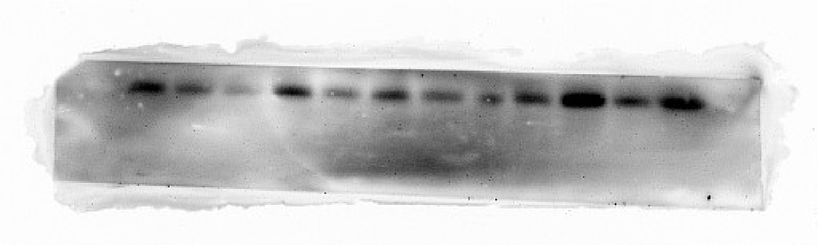

p21 (week 1)

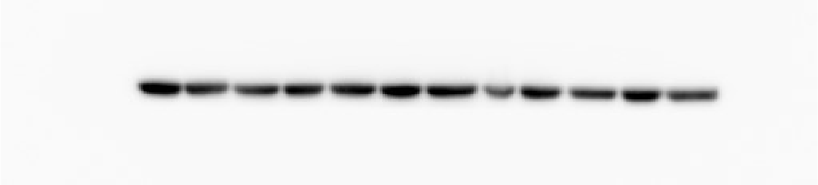

GAPDH (week 1)

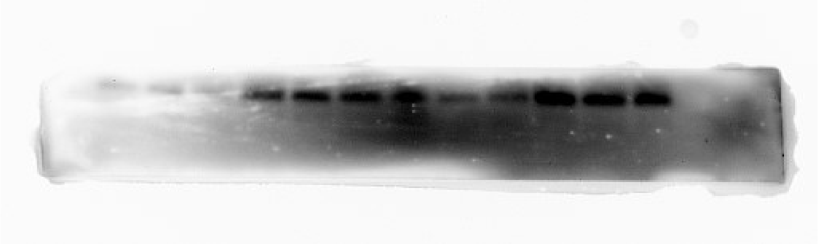

p21 (week 2)

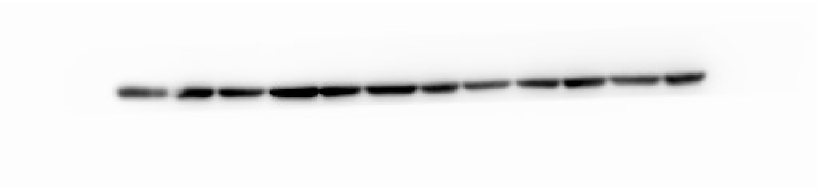

GAPDH (week 2)

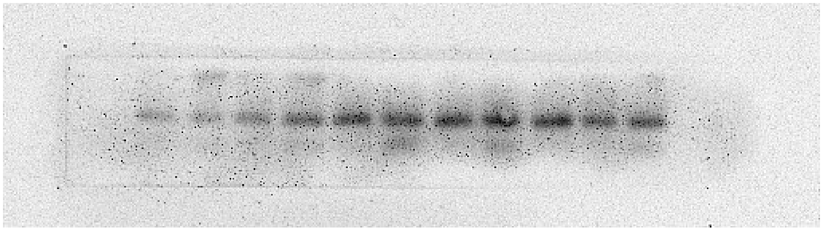

p21 (week 4)

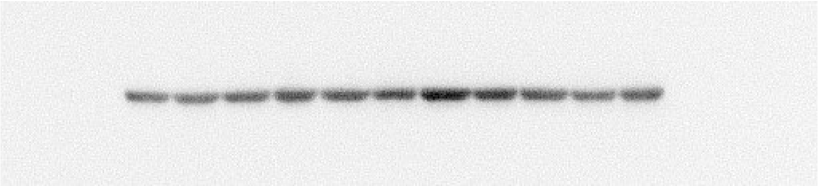

GAPDH (week 4)

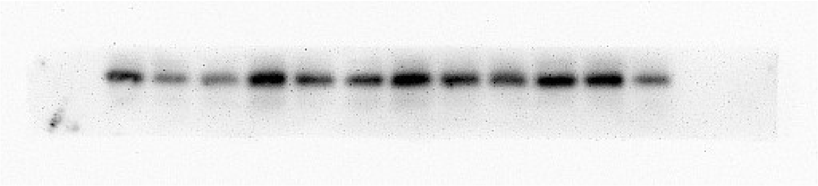

p21 (week 6)

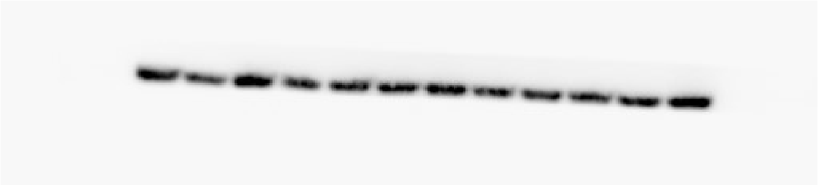

GAPDH (week 6)

Full Western blot images of Figure 5E

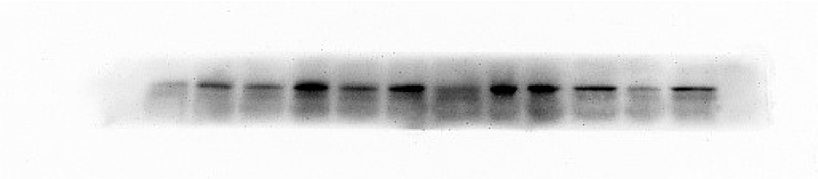

p53 (week 1)

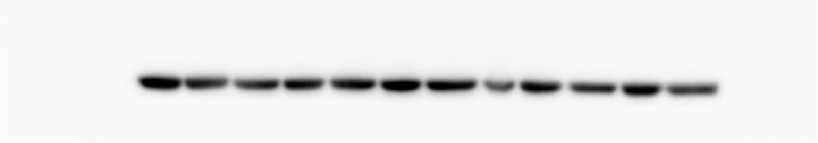

GAPDH (week 1)

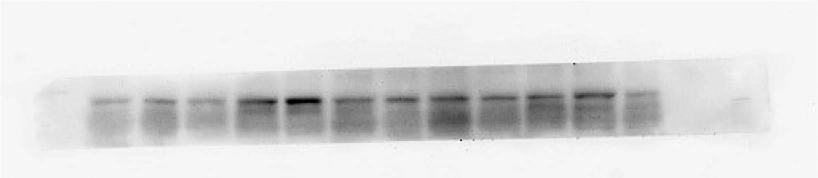

p53 (week 2)

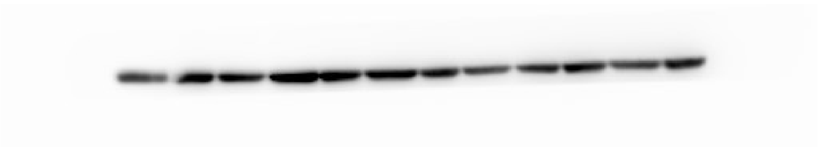

GAPDH (week 2)

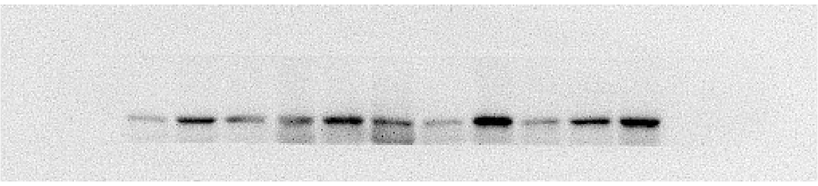

p53 (week 4)

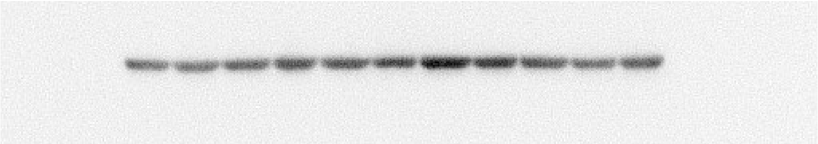

GAPDH (week 4)

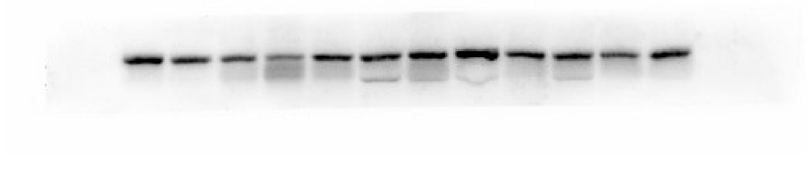

p53 (week 6)

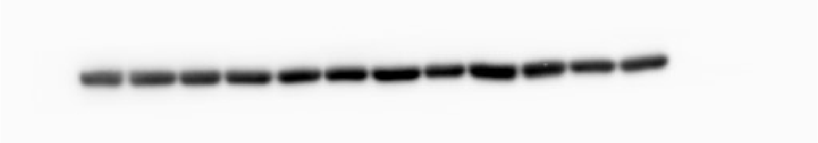

GAPDH (week 6)

Full Western blot images of Figure 5F

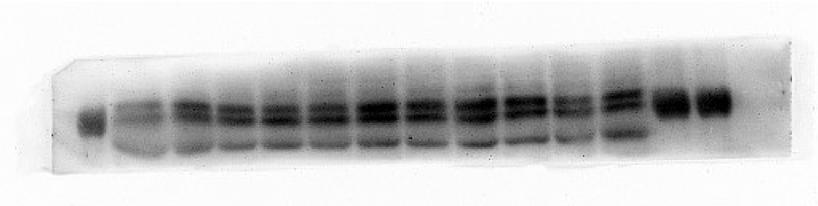

p-p53 (week 1)

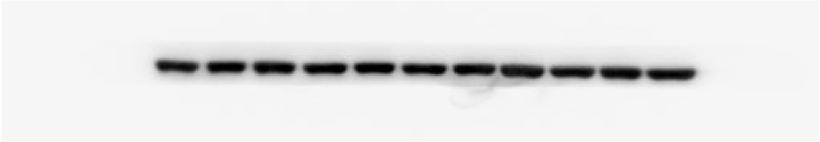

GAPDH (week 1)

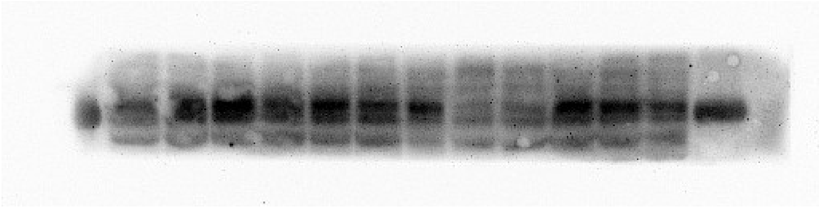

p-p53 (week 2)

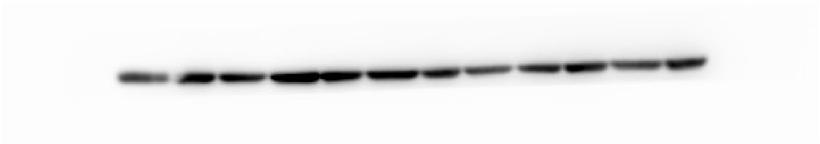

GAPDH (week 2)

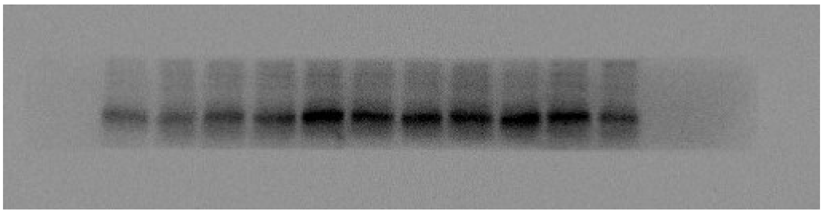

p-p53 (week 4)

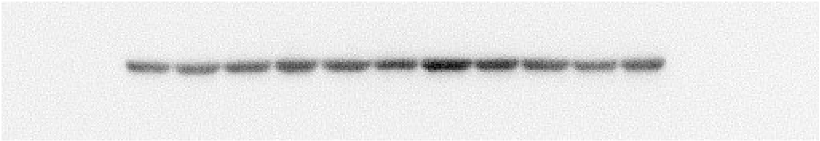

GAPDH (week 4)

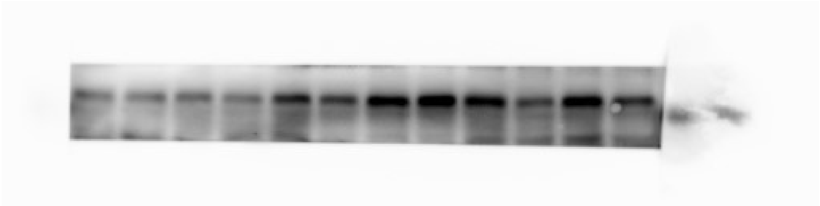

p-p53 (week 6)

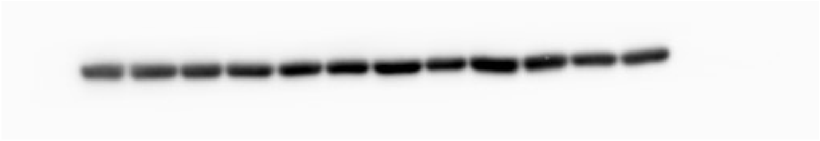

GAPDH (week 6)
